# A Novel Machine Learning Approach for Severity Classification of Diabetic Foot Complications Using Thermogram Images

**DOI:** 10.3390/s22114249

**Published:** 2022-06-02

**Authors:** Amith Khandakar, Muhammad E. H. Chowdhury, Mamun Bin Ibne Reaz, Sawal Hamid Md Ali, Serkan Kiranyaz, Tawsifur Rahman, Moajjem Hossain Chowdhury, Mohamed Arselene Ayari, Rashad Alfkey, Ahmad Ashrif A. Bakar, Rayaz A. Malik, Anwarul Hasan

**Affiliations:** 1 Department of Electrical Engineering, Qatar University, Doha 2713, Qatar; amitk@qu.edu.qa (A.K.); mkiranyaz@qu.edu.qa (S.K.); tawsifur.rahman@qu.edu.qa (T.R.); 2 Department of Electrical, Electronics and Systems Engineering, Universiti Kebangsaan Malaysia, Bangi 43600, Selangor, Malaysia; sawal@ukm.edu.my (S.H.M.A.); p113968@siswa.ukm.edu.my (M.H.C.); ashrif@ukm.edu.my (A.A.A.B.); 3 Department of Civil and Architectural Engineering, Qatar University, Doha 2713, Qatar; arslana@qu.edu.qa; 4 Technology Innovation and Engineering Education Unit, Qatar University, Doha 2713, Qatar; 5 Acute Care Surgery and General Surgery, Hamad Medical Corporation, Doha 3050, Qatar; rabdelmoaty@hamad.qa; 6 Weill Cornell Medicine-Qatar, Ar-Rayyan 24144, Qatar; ram2045@qatar-med.cornell.edu; 7 Department of Industrial and Mechanical Engineering, Qatar University, Doha 2713, Qatar; ahasan@qu.edu.qa

**Keywords:** diabetic foot, thermogram, thermal change index, machine learning, deep learning, diabetic foot, k-mean clustering, classical machine learning, deep learning, non-invasive diagnosis technique, diabetic foot severity classification

## Abstract

Diabetes mellitus (DM) is one of the most prevalent diseases in the world, and is correlated to a high index of mortality. One of its major complications is diabetic foot, leading to plantar ulcers, amputation, and death. Several studies report that a thermogram helps to detect changes in the plantar temperature of the foot, which may lead to a higher risk of ulceration. However, in diabetic patients, the distribution of plantar temperature does not follow a standard pattern, thereby making it difficult to quantify the changes. The abnormal temperature distribution in infrared (IR) foot thermogram images can be used for the early detection of diabetic foot before ulceration to avoid complications. There is no machine learning-based technique reported in the literature to classify these thermograms based on the severity of diabetic foot complications. This paper uses an available labeled diabetic thermogram dataset and uses the k-mean clustering technique to cluster the severity risk of diabetic foot ulcers using an unsupervised approach. Using the plantar foot temperature, the new clustered dataset is verified by expert medical doctors in terms of risk for the development of foot ulcers. The newly labeled dataset is then investigated in terms of robustness to be classified by any machine learning network. Classical machine learning algorithms with feature engineering and a convolutional neural network (CNN) with image-enhancement techniques are investigated to provide the best-performing network in classifying thermograms based on severity. It is found that the popular VGG 19 CNN model shows an accuracy, precision, sensitivity, F1-score, and specificity of 95.08%, 95.08%, 95.09%, 95.08%, and 97.2%, respectively, in the stratification of severity. A stacking classifier is proposed using extracted features of the thermogram, which is created using the trained gradient boost classifier, XGBoost classifier, and random forest classifier. This provides a comparable performance of 94.47%, 94.45%, 94.47%, 94.43%, and 93.25% for accuracy, precision, sensitivity, F1-score, and specificity, respectively.

## 1. Introduction

Diabetes mellitus (DM) is a chronic medical condition resulting from high amounts of sugar in the blood, which often leads to severe health complications such as heart-related diseases, kidney failure, blindness, and lower limb amputation [1]. Diabetes mellitus leads to foot ulcers, which might not heal properly due to poor blood circulation [2]. This might result in the spreading of infection and, eventually, can lead to amputation [3]. Amputations of lower limbs are more common in diabetic individuals, and according to statistics, diabetic foot lesions account for 25% of hospital admissions, and 40% of individuals presenting with diabetic foot necessitate amputation [4]. The reoccurrence rate of diabetic foot ulcers (DFUs) is also significantly high, with approximately 40% after the first year and 60% within three years of occurrence [5]. In an advanced and developed country such as Qatar, with more than 20% of the population being diabetic, it is reported that one of the most common reasons for people being admitted to hospitals is diabetic foot complications [6]. Every year in the USA alone, over one million diabetic patients suffer an amputation due to failure of recognizing and treating diabetic foot ulcers properly [7]. In Europe, 250,000 diabetic patients have their legs amputated with a death rate of 30% in one month and 50% in a year [8]. A diabetic patient under a ‘high risk’ category needs regular check-ups, hygienic personal care, and continuous expensive medication to avoid unwanted consequences. Diabetic foot ulcers lead to increased healthcare costs, decreased quality of life, infections, amputations, and death. Early detection and better DFU classification tools would enable a correct diagnosis, effective treatment, and timely intervention to prevent further consequences. 

Self-diagnosis at home, i.e., self-care such as monitoring without medical assistance, for early signs of ulcers could be useful in preventing severe after-effects. However, the easiest monitoring technique, visual inspection, has its limitations, such as people with obesity or visual impairment not being able to see their sites of ulcers easily. Some systems that allow for easy monitoring are TeleDiaFoS [9] and Bludrop [10]. TeleDiaFoS is a telemedicine system for the home telemonitoring and telecare of diabetic patients. In this system, the affected foot is scanned using a specialized patient module, which is operated with a simple two-button remote controller. While this method has shown great results that are comparable to Visitrak [11] and Silhouette [12], it requires a specialized module specifically for it. This might increase the cost. Bludrop has also introduced a patented system that promises high accuracy, but their system also requires specialized hardware. According to recent studies, a temperature monitoring system at home has been able to spot 97% of diabetic foot ulcers well in advance [13,14,15,16]. It has also been confirmed that patients going through the continuous monitoring of foot temperature had a low risk of foot complications [17]. There have been studies that have tried to find the relation between temperature and diabetes mortality [18,19], where they stressed that exposure to high and low temperatures increases the chances of diabetic mortality, especially in the aged population. Foltynski et al. in [20] concluded that ambient temperature does influence foot temperature even during foot ulceration and, thus, it should be taken into consideration, especially during the assessment for diabetic foot ulceration. As early as the 1970s, skin-temperature monitoring emerged as a useful tool for identifying patients at risk for ulceration. In the literature, the temperature monitoring approach uses the plantar foot temperature asymmetry between both feet, which is referred to as an “asymmetry analysis”, to find ulcers at an early stage [21]. A temperature difference of 2.22 °C (4 °F) over at least two consecutive days could be used as a threshold to indicate the starting time of preventive therapy for foot ulcers [14]. It has also been found that the system correctly identified 97% of observed DFUs, with an average lead time of 37 days [22]. Therefore, a foot with an elevated temperature (>2.2 °C compared to the opposite foot) can be considered to be “at-risk” of ulceration due to inflammation at the site of measurement.

Thermography is a popular technique used to examine thermal changes in a diabetic foot [22]. This is a pain-free, no-touch, and non-invasive technique. Several studies have proposed thermogram-based techniques for the study of diabetic feet [2,3,23], which include the identification of characteristic patterns in infrared images and the measurement of changes in thermal distribution. It was reported that the control group showed a specific butterfly pattern [24], while the DM group showed a large variety of spatial patterns [25,26]. It is possible to calculate and determine an estimation of thermal changes with respect to one foot as a reference [27,28,29,30] using the contralateral comparison of temperatures. However, if both feet have temperature changes, but neither of them has the butterfly pattern, one of the feet cannot act as a reference. Asymmetry cannot be measured even if there is a large temperature difference and identical spatial distributions present in both feet. An alternative approach is to calculate the temperature change using a control group butterfly pattern [31,32,33].

Machine learning (ML) techniques are gaining popularity in biomedical applications in assisting medical experts in early diagnosis [34,35,36]. The authors conducted an extensive investigation and developed a trained AdaBoost classifier, which achieved an F1-score of 97% in classifying diabetic and healthy patients using thermogram images [37]. However, the temperature distribution of a diabetic foot does not have a specific spatial pattern and it is, therefore, important to devise a method to distinguish the diabetic feet with different temperature distributions that do not depend on a spatial pattern. The spatial distribution may change after a while and the temperature changes are not significant in some cases. Moreover, the detection of irregular temperature rises in the plantar region is important for diabetic patients. The deep learning technique using thermogram images to classify the control and diabetic patients is not a well-studied domain. Moreover, the severity grading of diabetic foot is also lacking in the literature, which might help to establish an early warning tool for diabetic foot ulcer detection. A simple, effective, and accurate machine learning technique for diabetic foot severity stratification before foot ulcer development using thermogram images would be very useful.

Several studies [31,32,38,39,40,41,42,43,44,45] have attempted to extract features that can be used to identify the hot region in the plantar thermogram, which could be a sign of tissue damage or inflammation. In all of the works, the plantar region was split into six areas and different statistical features were extracted. In order to obtain various coefficients from texture and entropy characteristics, Adam et al. in [43] employed a discrete wavelet transformation (DWT) and higher-order spectra (HOS). In another work, the foot image was decomposed using a double-density dual-tree complex wavelet transform (DD-DT-CWT), and many features were retrieved from the decomposed images [42]. To categorize patients as normal or in the ulcer group, Saminathan et al. in [41] segmented the plantar area into 11 regions using region raising and retrieved texture characteristics. Maldonado et al. in [40] employed the DL approach to segment a visible foot picture, which was then used to segment the plantar area of the same patient’s thermogram image to identify ulceration or necrosis based on temperature differences. Hernandez et al. in [32] presented the thermal change index (TCI) as a quantitative indicator for detecting thermal changes in the plantar region of diabetic patients in comparison to a reference control group. They published a public database of diabetic foot thermogram pictures and named it the “Plantar Thermogram Database”, and used TCI to classify the participants into classes one to five based on the spatial temperature distribution and temperature range. The authors conducted considerable research and produced a trained AdaBoost classifier that classified diabetic and healthy patients using thermogram images with an F1-score of 97 percent [37]. Cruz-Vega et al. in [38] proposed a deep learning technique to classify the plantar thermogram database images in a non-convenient classification scheme, where the results were shown by taking two classes at a time and then averaging the results after ten folds of a different combination of two set classes. For the classification of class three and class four, a new diabetic foot thermogram network (DFTNet) was proposed, with a sensitivity and accuracy of 0.9167 and 0.853, respectively. However, the ground-truth classification of the patients in this database was performed using the TCI score, which takes the butterfly pattern of the control group which is individually used to compare each affected foot. Therefore, the classification entirely relies on the reliability of the TCI scoring technique, which was found questionable in two different aspects. Firstly, it was observed that thermogram images with a butterfly pattern even present in higher classes and the temperature distribution of severe diabetic patients as reported in other articles were incorrectly classified to lower classes based on the TCI score. Secondly, the state-of-the-art deep learning techniques failed to reliably classify the thermogram images into different classes, which were graded based on the TCI score. If a database is publicly available, it is easy to re-evaluate the labels in datasets if it is found that the labels are questionable [46]. Aradillas et al. [47] mentioned scenarios where they found errors in the labeling of the training samples in databases and proposed cross-validation techniques to remove them. Hu et al. [48] mentioned wrong labels in face-recognition databases and proposed a training paradigm that would take the dataset size into account and train itself by probabilistically removing some datasets that could have been incorrectly labeled. Ding et al. [49] proposed a semi-supervised two-stage approach in learning from noisy labels.

As per the above discussion, it can be seen that there is a lack of severity grading of the risk of ulcer development. The authors, including a set of expert medical doctors working in a diabetic foot clinic, stated that a foot plantar temperature distribution can help in the early detection of a foot ulcer development [50,51,52]. The scientific hypothesis of this work is whether machine learning approaches can be used to categorize thermograms into severity classes of patients with their risk of ulcer development. This severity classification can help in early treatment that could prevent diabetic foot ulcers. The improved performance of a machine learning model could help in early detection from the convenience of the patient’s home and reduce the burden on the healthcare systems, considering diabetic foot complications are very common and lead to expensive follow-up and treatment procedures throughout the world. All the above studies motivated the authors to carry out an extensive investigation on dataset re-labeling, using an unsupervised machine learning and pre-trained convolutional neural network (CNN) to extract features automatically from thermogram images, reduce feature dimensionality using the principal component analysis (PCA), and, finally, to classify thermograms using k-mean clustering [53] to revise the labels. The new labels identified by the unsupervised clusters were fine-tuned by a pool of medical doctors (MDs). Then, the authors investigated classical machine learning techniques using feature engineering and 2D CNNs with image enhancement techniques on the images to develop the best-performing classification network. The major contributions of this paper can be stated as follows:

The revision of labels of the thermogram dataset for the diabetic severity grading of a publicly available thermogram dataset using unsupervised machine learning (K-mean clustering), which is fine-tuned by medical doctors (MDs).

The extraction and ranking of relevant features from temperature pixels for classifying thermograms into diabetic severity groups.

The exploration of the effect of various image enhancement techniques on thermogram images in improving the performance of 2D CNN models in diabetic severity classification.

This work is the first of its kind to propose a machine learning technique to classify diabetic thermograms into different severity classes: mild, moderate, and severe.

The manuscript is organized into five sections, where Section 1 discusses the introduction and the related works and key contributions, Section 2 discusses the detailed methodology used in this work, and Section 3 and Section 4 presents the results and discussion. Finally, Section 5 presents the conclusions.

## 2. Methodology

**Figure 1:**
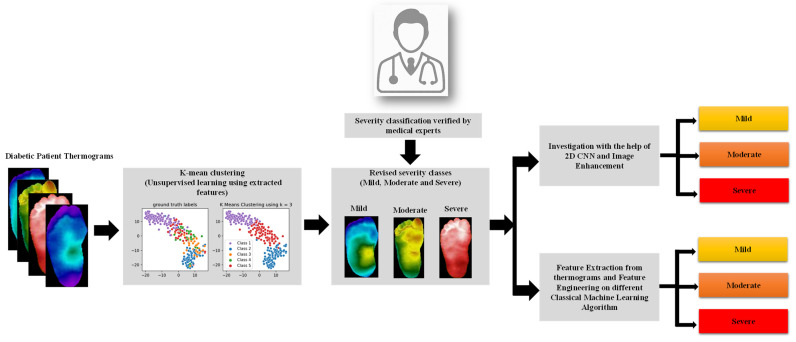
Illustration of the study methodology.

The methodology adopted in the work is presented in Figure 1. In the study, the thermogram was applied to a pre-trained CNN model to extract useful features, and then the feature dimensionality was reduced using principal component analysis (PCA), and the sparse feature space was then applied as an input to the k-mean clustering algorithm to provide unsupervised revised classes, which was then verified by medical doctors. The newly revised classes—mild, moderate, and severe—were then tested in terms of classification performance by 2D CNN approach using different image enhancement techniques and classical machine learning algorithms on the extracted features from the thermograms. The following sections went over the dataset used in the study, the K-mean clustering method used for unsupervised clustering, and the investigation performed using (i) thermogram images and 2D CNN networks and various image enhancement techniques, and (ii) classical machine learning algorithms with feature engineering (feature extraction, and feature reduction) on the thermogram images. This section also went through the performance indicators that were utilized to choose the best machine learning algorithm.

### 2.1. Dataset

In this study, 167 foot-pair thermograms of DM (122) and control (45) subjects were obtained from a public database from the General Hospital of the North, the General Hospital of the South, the BIOCARE clinic, and the National Institute of Astrophysics, Optics and Electronics (INAOE) for 3 years (from 2012 to 2014). All of these places were located in the city of Puebla, Mexico. The dataset had demographic information such as age (control group—27.76 ± 8.09; diabetic group—55.98 ± 10.57 years), gender, height, and weight. The participants who participated in the study were asked to lay in the supine position for 15 min to reach thermodynamic equilibrium to improve the accuracy of temperature variation due to diabetic complications and had avoided prolonged sun exposure, intense physical activity, and any effort that could affect blood pressure [32,39]. The participants were asked to remove their shoes and socks and clean their feet with a damp towel.

**Figure 2:**
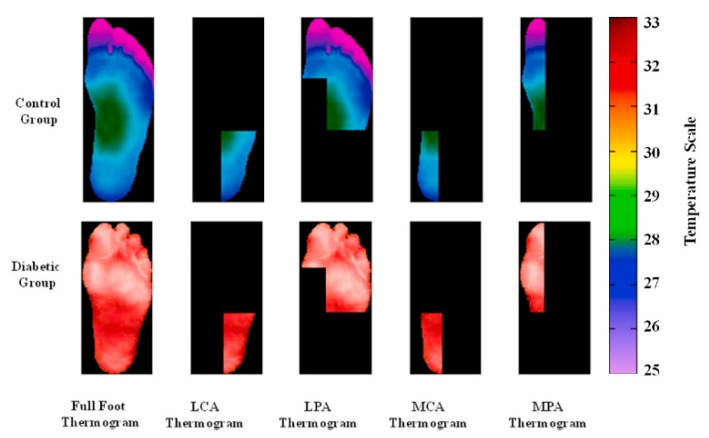
Sample of MPA, LPA, MCA, and LCA angiosomes of the foot for control and diabetic groups [37].

The data collection performed during resting time was to avoid exerting any effort that would impact the subjects’ blood pressure and, as a result, their plantar temperature. The dataset included segmented thermograms of the medial plantar artery (MPA), lateral plantar artery (LPA), medial calcaneal artery (MCA), and lateral calcaneal artery (LCA), a concept suggested by Taylor and Palmer [54], in addition to the segmented foot thermograms (Figure 2). Because it was utilized to compute local temperature distribution, the information gained utilizing angiosomes was related not only to the damage caused by DM in arteries, but also to the accompanying ulceration risk. The dataset, which is the largest diabetic thermogram public dataset, also included pixelated temperature measurements for complete feet and four angiosomes for both feet.

### 2.2. K-Mean Clustering Unsupervised Classification

In this part of the investigation, the underlying features of the images extracted using a pre-trained CNN model were used to cluster them in different groups using k-mean clustering. K-mean is the most popular method for clustering data in an unsupervised manner, and was proposed in 1967 [53]. K-mean is an unsupervised, non-deterministic, numerical, iterative method of clustering. In k-mean, each cluster is represented by the mean value of objects in the cluster. Here, we partitioned a set of n objects into k clusters, so that intercluster similarity was low and intracluster similarity was high. The similarity was measured in terms of the mean value of objects in a cluster. A similar concept was used in our investigation and could be divided into 4 steps:

- Pre-processing: Preparing the image so that it could be fed properly to the CNN model.
- Feature extraction: Using a pre-trained CNN model to extract the underlying features from a specific layer.
- Dimensionality reduction: Using principal component analysis (PCA) [55] to reduce the noise in the feature space and reduce the dimensionality
- Clustering: Using K-mean to cluster the images based on similar features.

In the pre-processing step, the image was first resized to 224 × 224 size. This was because the CNN model available in Keras [56], pretrained on ImageNet Dataset [57], required the input image to be of that size. Interpolation was applied to images before resizing to allow for rescaling by a non-integer scaling factor. This step did not change the properties of the image, and instead ensured that the image was formatted correctly.

**Figure 3:**
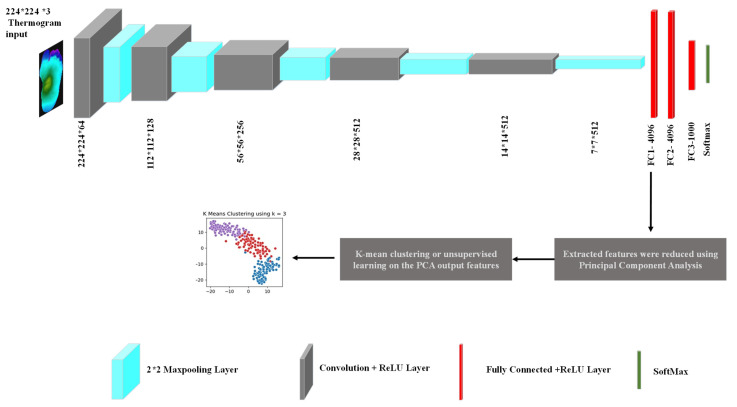
Detailed framework of thermogram image clustering.

**Figure 4:**
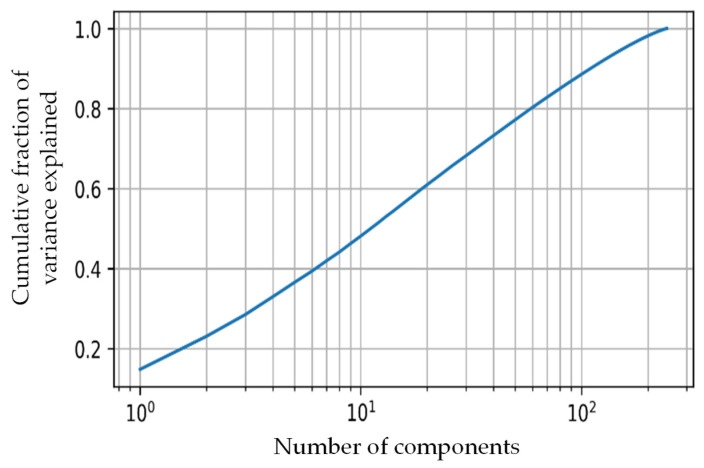
Cumulative variance vs. the number of PCA components.

For feature extraction in our study, the popular CNN network—VGG19 was used [58]. The VGG19 network developed by the Oxford Visual Geometry Group is a popular CNN for computer vision tasks because of its high performance and relative simplicity. The detailed framework, along with network architecture, can be seen in Figure 3. The pre-trained VGG19 model was used to extract 4096 features from the ‘FC1’ layer from each thermogram image. The selection of the network layer for feature extraction was a hyperparameter in this experiment, and it was tuned to obtain the parameter that provided the best result, which is a common exercise. We empirically found out that the FC1 layer gave us the best result similar to other studies that investigated k-means clustering [59,60]. The number of features needed to be reduced so that the k-mean algorithm did not overfit and it remained robust to noise. PCA was used to transform and reduce the features such that only the most important features were retained [51]. Figure 4 shows the cumulative variance versus the number of components after PCA. It can be seen that the first 10 components provided a cumulative variance of 0.5, and after adding 90 more components, the variance rose to 0.9. Thus, significant information was retained in the initial components. In our study, we used 45 components, which helped to achieve 75% cumulative variance, which is usually an acceptable threshold in various k-mean clustering studies [61,62,63,64].

The reduced and transformed features were separated into different groups using k-mean clustering [65]. This is an unsupervised machine learning algorithm, which popularized dealing with non-labeled data [66,67]. K-mean’s aim was to group data that were similar in feature space. The algorithm flow can be seen in Algorithm 1 below. In our study, max_iter was set to 500 and the break condition that happened only after line 4 was fulfilled for all clusters.
**Algorithm 1:** K-mean clustering**Input**:Feature matrix, number of centroids (*k*)**Output**:Trained model
1:**for**i←1 to max_iter
**do**
2:Assign each point with the centroid that it is closest to in latent space;
3:Recalculate the position of the clusters (centroid_position) to be equal to the mean position of all of its associated points;
4:**if**$centroid\_position_{i}==centroid\_position_{i-1}$**then**
5:**break**;
6:i++;
7:**end for**

The algorithm aimed to reduce inertia. Inertia is the sum of squared Euclidean distances from a centroid to its associated data points and can be seen mathematically in Equation (1).

(1) $$Inertia=\overset{k}{\underset{j=1}{\sum }}\overset{n}{\underset{i=1}{\sum }}∥x_{i}^{\left(j\right)}-c_{j}∥{}^{2}$$

where *k* is the total number of clusters, *n* is the number of samples associated with a cluster, *x* is the position of the sample in the feature space, and *c* is the position of the centroid in the feature space.

K-means++ [68] was used to initialize the centroid position. This method helped to achieve good clustering performance and reduced computational complexity. In this method, the first centroid was selected from the data with uniform probability. The other centroids were selected from the data with a probability proportionate to their distance from the nearest centroid. Hence, the initial centroids were close to the data points but were far apart from each other.

As stated earlier, the labeled dataset provided by k-mean clustering was used for the investigation using (i) transfer learning, image enhancement, and transfer learning 2D CNN and (ii) classical machine learning techniques and feature engineering on the extracted features from the thermograms.

### 2.3. Two-Dimensional CNN-Based Classification

Two-dimensional CNN is widely used in biomedical applications for automatic and early diagnosis of anomalies such as COVID-19 pneumonia, tuberculosis, and other diseases [69,70]. A labeled dataset can be divided into training and testing datasets, with the training dataset being used to train the network and the unseen testing dataset being used to verify its performance. During the training process, a portion of the training dataset is used for validation to avoid overfitting. Five-fold cross-validation was employed in this study, which divided the dataset five-fold and found the performance metric for the testing dataset five times. Each time, one of the folds was used as testing dataset, and the remaining folds were used for training and validation. This approach helped in stating results considering the complete dataset and making sure the test data were always unseen. The final results were the overall and weighted result of the five folds; detailed performance metric was shown later. As larger amount of data used for training always helps in obtaining better trained model, the authors used popular augmentation techniques (rotation and translation) to increase the training data size. The rotation operation used for image augmentation was performed by rotating the images in clockwise and counter-clockwise directions with angles from 5 to 30 in increments of 2°. Image translation was conducted by translating images horizontally and vertically from −15 to 15%. The details of the training, validation and testing dataset for 2D binary and severity classifier are shown in Table 1.

*Transfer learning:* As we had a limited dataset, which can be seen in Table 1, we could make use of pre-trained models which were trained on a large ImageNet database [71]. These pre-trained networks were trained on very large ImageNet database [71], and had good classification performance. These networks could be further trained on any other classification problem, and this is known as transfer learning. Based on extensive literature review and previous performances [37], in this study, seven well-known pre-trained deep CNN models were used for thermograms’ classification: ResNet18, ResNet50, ResNet100 [72], DenseNet201 [72], InceptionV3 [73], VGG19 [58], and MobileNetV2 [74].

**Figure 5:**
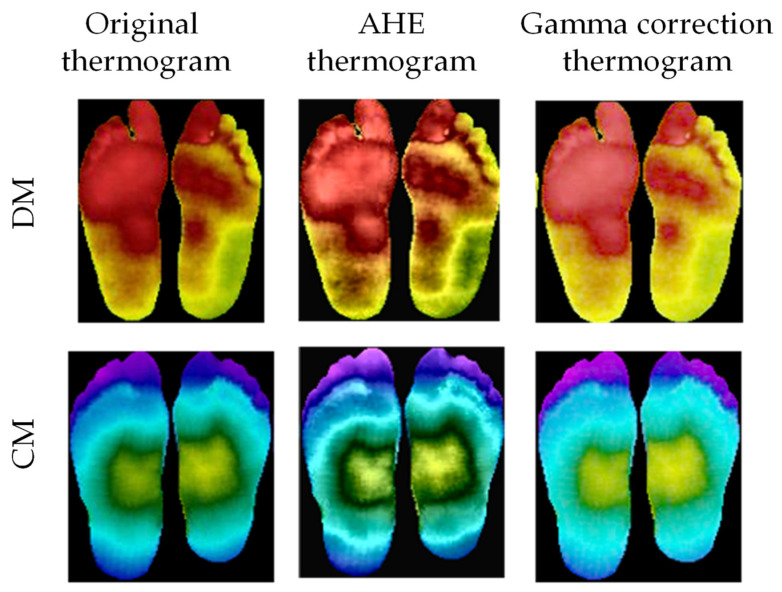
Original thermogram versus enhanced thermogram using AHE and gamma correction for DM and CG [37].

*Image enhancement:* It was found that image enhancement techniques such as adaptive histogram equalization (AHE) [75] and gamma correction [70,76] can help the 2D CNN in improving its classification performance for thermograms [37]. Some samples of the image enhancement on DM and CG can be seen in Figure 5. The authors investigated the improvement of performance made possible by the different image enhancement techniques, and they were reported in the Experimental Results section.

### 2.4. Classical Machine Learning Approach

This section discusses the features extracted for classical ML techniques, feature reduction techniques, feature ranking techniques, machine learning classifiers, and the extensive investigations performed using two approaches.

#### 2.4.1. Extracted Features and Feature Reduction

The authors looked through the literature carefully to summarize the features that are used in clinical practice and machine learning approaches to analyze the foot thermograms for diabetic foot diagnosis. The details of the final list of features identified by the authors were mentioned in their previous work in [37] and also mentioned below:

(2) $$Thermal\;Change\;Index\;\left(TCI\right)=\frac{CG_{ang}-DM_{ang}}{4}$$

where *CG_ang_* and *DM_ang_* are the temperature values of the angiosome for the control group and a *DM* subject, respectively.

(3) $$Estimated\;Temperature\;\left(ET\right)=\frac{a_{j-1}C_{j-1\;}+a_{j}C_{j}+a_{j+1}C_{j+1}}{a_{j-1}+a_{j}+a_{j+1}}$$

(4) $$Estimated\;Temperature\;Difference\;\left(ETD\right)=|ET_{left\;Angiosome}-ET_{right\;angiosome}|$$

(5) $$Hot\;Spot\;Estimator\;\left(HSE\right)=|c_{l}-ET|$$

A histogram for the percentage of pixels in the thermogram (either complete foot or angiosomes) in the different classmark temperatures (*C*_0_ = 26.5 °C, *C*_1_ = 28.5 °C, *C*_2_ = 29.5 °C, *C*_3_ = 30.5 °C, *C*_4_ = 31 °C, *C*_5_ = 32.5 °C, *C*_6_ = 33.5 °C, and *C*_7_ = 34.5 °C) was generated to equate the parameters in Equations (3)–(5). The classmark temperature and the associated percentage of pixels in that region were denoted by the terms *C_j_* and *a_j_*, respectively. The percentage of pixels in the surrounding classmark temperatures *C_j−1_* and *C_j+1_* were represented by the values *a_j−1_* and *a_j+1_*, respectively.

In addition to these parameters, the authors formulated *NRTclass j*, which was the number of pixels in the class j temperature range over the total number of non-zero pixels, where class *j* could be class 1 to 5. This parameter is visually very important for distinguishing the variation in the plantar temperature distribution, and was also reported in the authors’ previous work [37].

Thus, TCI, highest temperature value, NTR (class 1–5), HSE, ET, ETD, mean, median, SD of temperature for the distinct angiosomes, LPA, LCA, MPA, MCA, and full foot were among the 37 features that could be employed for early diabetic foot identification. In their prior study [37], the authors published the statistics of the data provided by the source [39].

By determining the association between the various features, the final list of features was streamlined to eliminate redundant features. Features with a correlation of greater than 95% were deleted, improving overall performance by lowering the number of redundant features and preventing overfitting [77,78].

#### 2.4.2. Machine Learning Classifiers

The authors also explored different machine learning (ML) classifiers in this study to compare the performances. The popular ML classifiers used in the study were multilayer perceptron (MLP) [79], support vector machine (SVM) [80], random forest [81], extra tree [82], GradientBoost [83], logistic regression [84], K-nearest neighbor (KNN) [85], XGBoost [86], AdaBoost [87], and linear discriminant analysis (LDA) [88].

#### 2.4.3. Classical Machine Learning Approach 1: Optimal Combination of Feature Ranking, Number of Features

After optimization, the dataset’s shortlisted parameters were evaluated to determine decisions and the best features for severity classification. The multi-tree extreme gradient boost (XGBoost) [89], random forest [90], extra tree [91], chi-squared [92], pearson correlation coefficient [93], recursive feature elimination (RFE) [94], logistic regression [95], and LightGBM [96] were used to identify three different sets of feature ranking. Rigorous research determined the greatest combination of features that offered the best performance, and the best-performing top-ranked features from the different feature-ranking methodologies were used to identify the best combination of features.

#### 2.4.4. Classical Machine Learning Approach 2: Stacking-Based Classification

**Figure 6:**
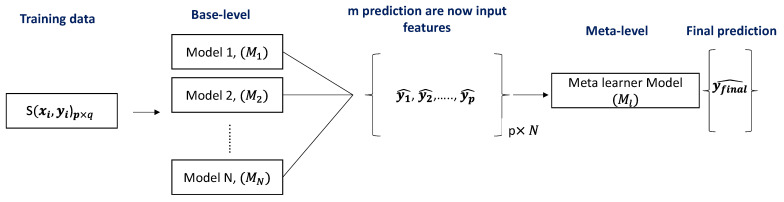
Schematic representation of the stacking model architecture.

We presented a stacking-based classifier that worked by merging numerous best-performing classifiers created by different learning algorithms $L_{1},\ldots \ldots ,L_{N}$ on a single dataset S, which consisted of examples $s_{i}=\left(x_{i},y_{i}\right)$, i.e., pairs of feature vectors ($x_{i}$) and their classifications ($y_{i}$). A collection of base-level classifiers $M_{1},\ldots \ldots ,M_{N}$ was trained in the first phase, where $M_{i}=L_{i}\left(S\right)$. A meta-level classifier was trained in the second step, which integrated the outputs of the base-level classifiers (Figure 6).

A cross-validation approach was used to build a training set for learning the meta-level classifier. We applied each of the base-level learning algorithms to four folds of dataset for five-fold cross-validation, leaving one fold for testing. A probability distribution over the possible class values was predicted by each base-level classifier. When applied to example *x*, the prediction of the base-level classifier *M* was a probability distribution:

(6) $$P^{M}\left(x\right)=\left(P^{M}\left(c_{1}|x\right),P^{M}\left(c_{2}|x\right),\ldots \ldots .,P^{M}\left(c_{m}|x\right)\right),$$

$P^{M}\left(c_{i}|x\right)$ signifies the probability that example *x* belonged to class $c_{i}$ as estimated (and predicted) by classifier *M*, where $\left(c_{1},c_{2},\ldots \ldots ,c_{m}\right)$ is the set of possible class values. Classifier *M* predicted the class $c_{j}$ with the highest class probability $P^{M}\left(c_{j}|x\right)$. The meta-level characteristics are the probabilities predicted by each of the base-level classifiers for each conceivable class, i.e., $P^{M_{j}}\left(c_{i}|x\right)$ for *i* = 1……*m* and *j* = 1……*N*, where $\left(c_{1},c_{2},\ldots \ldots ,c_{m}\right)$ is the set of possible class values and $P^{M}\left(c_{i}|x\right)$ denotes:

### 2.5. Performance Evaluation and Classification Scheme

In all of our experiments, we reported sensitivity, specificity, precision, accuracy, *F*1-score, and area under the curve (AUC) for five folds, as our evaluation metrics.

(7) $$Sensitivity=\frac{\left(TP\right)}{\left(TP+FN\right)}$$

(8) $$Specificity=\frac{\left(TN\right)}{\left(TN+FP\right)}$$

(9) $$Precision=\frac{\left(TP\right)}{\left(TP+FP\right)}$$

(10) $$Accuracy=\frac{TP+TN}{\left(TP+FN\right)+\left(FP+TN\right)}$$

(11) $$F1\;Score=\frac{\left(2*Precision*Sensitivity\right)}{\left(Precision+Sensitivity\right)}$$

Here, *TP*, *FP*, *TN*, and *FN* are true positive, false positive, true negative, and False negative. In any classification, *TP* is the number of correctly identified thermograms, *TN* is the number of correctly identified thermograms of the other class, *FP* is the number of thermograms misclassified and *FN* is the number of thermograms of the other class misclassified. In this paper, weighted performance metric with 95% confidence interval was reported for sensitivity, specificity, precision, and *F*1-score, and for the accuracy, the overall accuracy was reported.

All the experiments were done on google colab platform and used GPU GeForce RTX2070 Super which is manufactured by NVIDIA, it is located in Santa Clara, CA, USA. The other software was Matlab from Mathworks, Natick, MA, USA.

## 3. Experimental Results

This section of the paper provides the results of the various important experiments of the paper. The details are below:

### 3.1. K-Mean Clustering Unsupervised Classification

**Figure 7:**
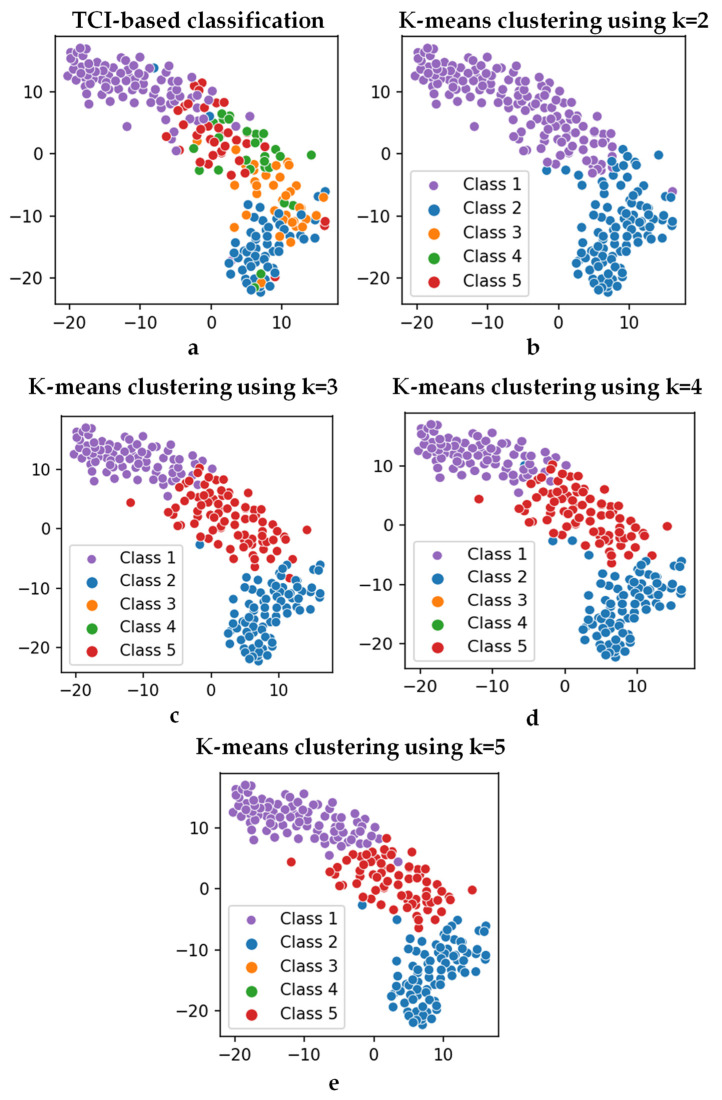
t-SNE plots with the (**a**) TCI-based classes (class 1–class 5) and output from k-mean clustering with (**b**) k = 2, (**c**) k = 3, (**d**) k = 4, and (**e**) k = 5.

As stated in the previous section, k-mean clustering was used to classify the thermogram images in an unsupervised fashion. This was conducted by using the features extracted from the VGG-19 network, which were later reduced and transformed using PCA. K-mean clustering was applied for various cluster numbers, i.e., k in Algorithm 1. The optimal number of clusters would be equivalent to the correct severity class of the thermograms, later verified by medical experts. The T-distributed stochastic neighbor embedding (t-SNE) plots, which are very useful in visualizing the clustering of data in two dimensions [97,98], for various clusters can be seen in Figure 7. The t-SNE is a dimensionality reduction method that is used to visualize high-dimensional data. In this work, the high-dimensional feature space was visualized by reducing it to two-dimensions using t-SNE, and then plotting it in a scatterplot. It was evident that there was a clear distinction in clustering for cluster sizes two and three compared to a cluster size of five (original classification performed using TCI scores, i.e., class one–class five). As stated in Figure 4, 45 components were used in the k-mean clustering.

**Figure 8:**
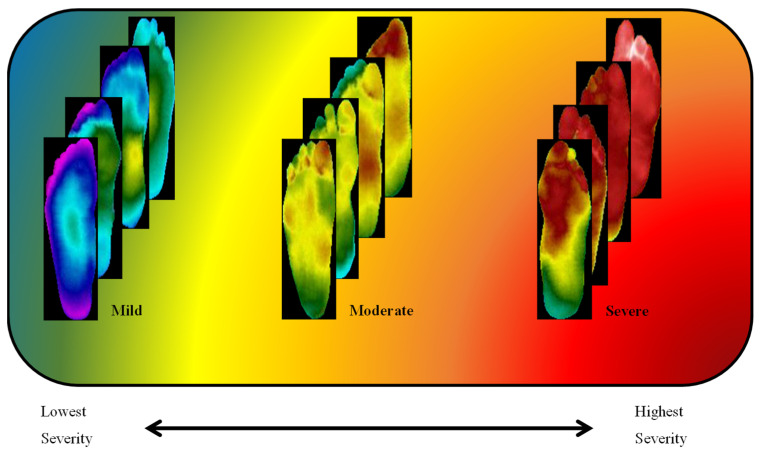
Diabetic thermograms classified into three severities: mild, moderate, and severe.

The clusters found using the k-mean clustering approach were confirmed by experts in the diabetic foot complication domain as thermograms of patients with mild, moderate, and severe severity, and can be seen in Figure 8. The experts used their experience with plantar foot temperature distribution amongst diabetic patients as reference for confirming the severity classification. The clusters found by k-mean clustering provided three differentiable sets of thermograms. Plantar foot thermograms with slightly a deviated butterfly pattern were labelled as mild by medical experts. The second set of plantar foot thermograms where there was an abnormal and high-temperature distribution was labelled as moderate by medical experts, as the temperature distributions were still not very high (which is usually indicated by the red color distribution in thermograms) and could indicate the possibility of ulcer development in the near future. The third set of plantar foot thermograms had extreme high temperature (which is usually indicated by the red color distribution in thermograms) throughout the foot, indicating the possibility of ulcer development in the immediate future, and were labelled as severe. Such clustering was not found in the original classification using the TCI-based method (i.e., class one–class five). The clustering algorithm separated the images into three clusters. The mild class had 82 images, the moderate class had 84 images, and the severe class had 78 images.

**Figure 9:**
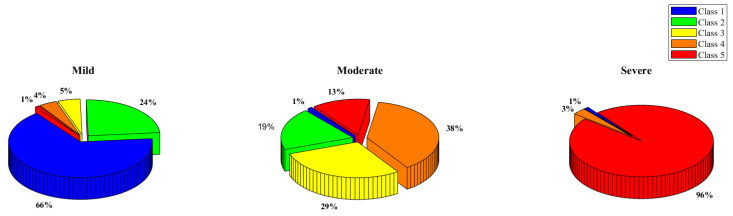
Class 1, 2, 3, 4, and 5 distribution in the K-mean clustering categories—mild, moderate, and severe.

The output of the k-mean clustering could be further understood with the help of the pie charts in Figure 9, which showcase the distribution of the classes in the different clusters found by the k-mean clustering approach. It can be clearly seen that many class one thermograms were very similar to class five thermogram (refer to the severe class in the pie chart) and many class three, class four, and class five were similar to class one and class two (refer to the mild class in the pie chart).

### 3.2. Classical Machine Learning-Based Classification

Once the new labeled datasets were confirmed using the k-mean clustering approach and verified by the medical experts, the highlighted correlated features from the 37 extracted were reduced to 27 features—NRT (class one), NRT (class two), NRT (class three), NRT (class four), NRT (class five), highest temperature, TCI, HSE, ETD, mean, and STD of MPA angiosome; HSE, ET, ETD, mean, and STD of LPA angiosome; HSE, ET, ETD, mean, and STD of LCA angiosome; HSE, ETD, and STD of MCA angiosome; HSE, ETD, and STD of full feet. The heatmaps of the correlation matrix with all features and after removing the highly correlated features are shown in Supplementary Figure S1.

#### 3.2.1. Classical Machine Learning Approach 1: Optimal Combination of Feature Ranking, Number of Features

In this experiment, 3 feature selection techniques with 10 machine learning models were investigated with 27 optimized features to identify the best-combined results in 810 investigations. The best-performing combination is presented in Table 2. It can be seen that the XGboost classifier with the random forest feature selection technique and the top 25 features showed the best performance of 92.63% weighted F1-score in the diabetic severity classification.

#### 3.2.2. Classical Machine Learning Approach 2: Stacking-Based Classification

As stated earlier, we shortlisted the major contributing features from the 29 finalized features after a feature reduction. The shortlisting was performed based on finding the features which were categorized as highly correlated with the output based on all the techniques—Pearson, Chi-square, RFE, logistic, random forest, and LightGBM. Table 3 showcases the features which had a total of more than four, and it was found that only eight features (TCI, NRT (class four), NRT (class three), the mean of MPA, mean of LPA, ET of LPA, mean of LCA, and highest temperature) were top ranked features by all the techniques (Pearson, Chi-Square, RFE, logistics, random forest, and LightGBM). These eight features were used to check the performance using different machine learning classifiers and stacking performed on the top three classifiers, providing the best result, as can be seen in Table 4. The stacking classifier created using the trained gradient boost classifier, XGBoost classifier, and random forest classifier was the best-performing classifier, with 94.47%, 94.45%, 94.47%, 94.43%, and 93.25% for accuracy, precision, sensitivity, F1-score, and specificity, respectively. The performance of this approach was better than Approach 1, as shown in Table 2, with the only difference in the inference time, which was found more in the stacking classifier, as expected.

### 3.3. Two-Dimensional CNN-Based Classification

**Figure 10:**
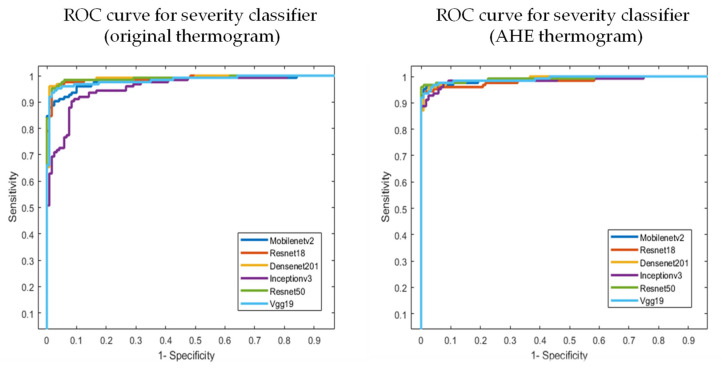
AUC for the original and best-performing AHE thermogram in severity classification.

As discussed earlier, the authors investigated state-of-the-art transfer learning networks—ResNet18, ResNet50, VGG19, DenseNet201, InceptionV3, and MobileNetv2, along with popular image enhancement techniques. The best-performing network and enhancement types (e.g., AHE) were used for severity classification. Gamma correction did not improve the performance, as it did not help in sharpening the distinguishing features. Further discussion on the image enhancement techniques was provided in the Discussion section. Independent foot images were used to check if the different pre-trained networks could classify them into different severity levels. Table 5 reports the best-performing AHE, with VGG19 showcasing the best performance. It is to be noted that the improved performance came with a tradeoff of a slightly higher inference time. The increase in inference time to classify the thermogram image was caused due to the addition of the image enhancement technique as a pre-processing step. However, the increase in inference time due to this additional pre-processing step was only 1 ms, which would not affect even real-time classification applications. It is more important to have improved performance than to have a faster response. In our study, the image enhancement helped in the performance with a 1 ms excess delay. The ROC curves for the original and AHE thermograms are also shown in Figure 10.

## 4. Discussion

As mentioned earlier, the authors conducted an extensive investigation and developed a trained AdaBoost classifier to achieve an F1-score of 97% in classifying diabetic and healthy patients using thermogram images [37]. The diabetic foot thermogram network (DFTNet) proposed a deep learning technique to classify the images of the plantar thermogram database in a non-convenient classification scheme, where the results were shown by taking two classes at a time and then averaging the results after ten folds of a different combination of two set classes [38]. As discussed earlier, the ground-truth classification of the patients in this database was performed using the TCI score and entirely relied on the reliability of the TCI scoring technique. Previous works and the lack of severity grading of diabetic foot complications motivated this work.

**Figure 11:**
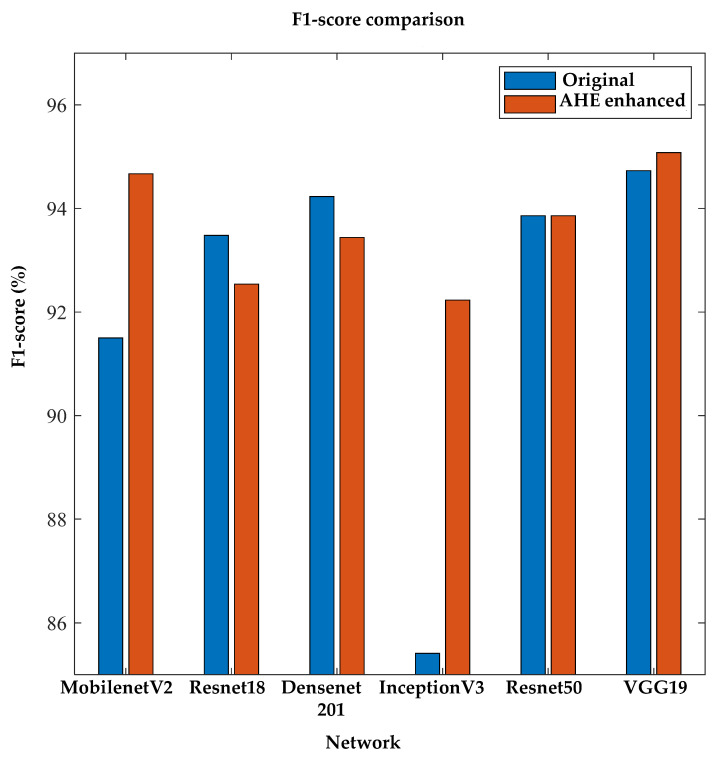
Comparison of F1-score between original and AHE-enhanced thermogram images using 2D severity classifier.

Again, to the best of the author’s knowledge, no previous studies have investigated image enhancement techniques for diabetic foot severity classification using thermograms for 2D CNNs. The authors investigated different pre-trained networks and also found that the image enhancement techniques helped in the classification performance. Figure 11 confirms the improved performance of the image enhancement technique using the F1-score performance metric.

Even the classical machine learning approach provided better classification performance using TCI, NRT (Class 4), NRT (Class 3), the mean of MPA, mean of LPA, ET of LPA, mean of LCA, and highest temperature extracted features from the thermogram. The stacked classifiers using popular gradient boost, XGBoost, and random forest classifiers provided a comparable classification performance with a much lower inference time (Table 4 and Table 5). The following interesting results could be summarized:

AHE, due to its special equalization, helped with the severity classification using the pre-trained VGG 19 network.

A deeper layer network such as DensetNet201 did not improve the performance for image enhancement, which could be attributed to the simplistic nature of the thermogram images, which did not require a very deep network to extract meaningful features.

The proposed features [37] and new classification technique helped in classifying the diabetic thermograms into different severity groups.

## 5. Conclusions

Diabetic foot problems are an issue that has great consequences not only in terms of mortality, but also in terms of the expense needed for monitoring and controlling the disease. Thus, early detection and severity classification can help in preventing such complications. The deployment of machine learning in biomedical applications could help in preparing easy-to-use solutions for early detection, not only for medical experts to save their time, but also since such solutions can be useful for patients in a home setting. They can use it in their homes, especially during pandemic times, where visits to healthcare services are preferred to be limited, avoiding stress on the healthcare system. The authors in the paper proposed a novel framework to cluster diabetic thermograms based on severity, which was not present in the literature, and the ones that were available needed verification. The trained 2D CNN and classical machine learning models could help in severity stratification using foot thermograms, which can be captured using infrared (IR) cameras. To the best of the author’s knowledge, this was the first study analyzing such a diabetic foot condition severity classifier for the early and reliable stratification of diabetic foot. The machine learning classifier performance was comparable to the 2D CNN performance using image enhancement. In conclusion, such a system could be easily deployed as a web application, and patients could benefit from remote health care using just an infrared camera and a mobile application, which is a future direction of our research.

## Figures and Tables

**Table 1:** Details of the dataset used for training (with and without augmentation), validation, and testing.

Classifier	Dataset	Count of Diabetic Thermograms/Cluster Identified in the Paper	Training Dataset Details			
			Training (60% of the Data) Thermogram /Fold	Augmented Train Thermogram /Fold	Validation (20% of the Data) Thermogram /Fold	Test (20 % of the Data) Image/Fold
Severity	Contreras et al. [39]	Mild	43	2040	3	11
		Moderate	48	2244	4	11
		Severe	93	1806	7	24

**Table 2:** Performance metrics for the best-performing combinations using Approach 1.

Classifier	Feature Selection	# of Feature	Class	Accuracy	Precision	Sensitivity	F1-Score	Specificity	Inference Time (ms)
XGBoost	Random Forest	25	Mild	92.59 ± 6.80	91.53 ± 7.23	94.74 ± 5.80	93.1 ± 6.58	91.94 ± 7.07	0.397
			Moderate	92.59 ± 6.47	86.15 ± 8.53	88.89 ± 7.76	87.5 ± 8.17	93.89 ± 5.91	
			Severe	92.59 ± 4.61	96.64 ± 3.17	93.50 ± 4.34	95.04 ± 3.82	91.67 ± 4.86	
			Overall	92.59 ± 3.29	92.72 ± 3.26	92.59 ± 3.29	92.63 ± 3.28	92.31 ± 3.34	

**Table 3:** Shortlisted features based on the six feature ranking techniques.

Feature	Pearson	Chi-Square	RFE	Logistics	Random Forest	LightGBM	Total
**TCI**	**√**	**√**	**√**	**√**	**√**	**√**	6
**NRT (Class 4)**	**√**	**√**	**√**	**√**	**√**	**√**	6
**NRT (Class 3)**	**√**	**√**	**√**	**√**	**√**	**√**	6
**Mean of MPA**	**√**	**√**	**√**	**√**	**√**	**√**	6
**Mean of LPA**	**√**	**√**	**√**	**√**	**√**	**√**	6
**ET of LPA**	**√**	**√**	**√**	**√**	**√**	**√**	6
**Mean of LCA**	**√**	**√**	**√**	**√**	**√**	**√**	6
**Highest Temperature**	**√**	**√**	**√**	**√**	**√**	**√**	6
**NRT (Class 2)**	**√**	**√**	**√**	**√**	**√**		5
**NRT (Class 1)**	**√**	**√**	**√**	**√**	**√**		5
**ET of LCA**	**√**	**√**	**√**	**√**	**√**		5
**NRT (Class 5)**	**√**	**√**		**√**	**√**		4
**STD of MPA**	**√**	**√**	**√**	**√**			4
**ETD of MPA**		**√**	**√**	**√**		**√**	4
**STD of MCA**	**√**	**√**	**√**	**√**			4
**ETD of MCA**	**√**	**√**		**√**		**√**	4
**STD of LPA**	**√**	**√**	**√**	**√**			4
**HSE of LCA**			**√**	**√**	**√**	**√**	4
**ETD of Full foot**		**√**	**√**	**√**		**√**	4

**Table 4:** Class-wise performance metrics for the top 3 machine learning classifiers and the stacked classifier.

Classifier	Class	Accuracy	Precision	Sensitivity	F1-Score	Specificity	Inference Time (ms)
Gradient Boost	**Mild**	92.01 ± 4.98	91.38 ± 5.15	92.98 ± 4.69	92.17 ± 4.93	91.71 ± 5.06	0.379
	**Moderate**	92.01 ± 4.73	84.50 ± 6.32	86.51 ± 5.97	85.49 ± 6.15	93.92 ± 4.17	
	**Severe**	92.01 ± 3.37	96.30 ± 2.35	94.35 ± 2.87	95.32 ± 2.63	89.58 ± 3.80	
	**Overall**	92.01 ± 3.40	92.10 ± 3.38	92.01 ± 3.40	92.04 ± 3.40	91.20 ± 3.55	
XGBoost	Mild	93.24 ± 4.61	90.08 ± 5.49	95.61 ± 3.76	92.77 ± 4.76	92.51 ± 4.83	0.336
	Moderate	93.24 ± 4.38	89.26 ± 5.41	85.71 ± 6.11	87.45 ± 5.78	95.86 ± 3.48	
	Severe	93.24 ± 3.13	96.75 ± 2.21	95.97 ± 2.45	96.36 ± 2.33	90.42 ± 3.66	
	Overall	93.24 ± 3.15	93.26 ± 3.15	93.24 ± 3.15	93.22 ± 3.15	92.31 ± 3.34	
Random Forest	Mild	91.80 ± 5.04	89.19 ± 5.7	86.84 ± 6.21	88.00 ± 5.97	93.32 ± 4.58	0.327
	Moderate	91.80 ± 4.79	90.43 ± 5.14	82.54 ± 6.63	86.31 ± 6.00	95.03 ± 3.80	
	Severe	91.80 ± 3.41	93.51 ± 3.07	98.79 ± 1.36	96.08 ± 2.42	84.58 ± 4.49	
	Overall	91.80 ± 3.44	91.71 ± 3.46	91.80 ± 3.44	91.67 ± 3.47	89.32 ± 3.88	
Stacking (Gradient Boost + XGBoost + Random Forest)	Mild	94.47 ± 4.20	91.53 ± 5.11	94.74 ± 4.10	93.10 ± 4.65	94.39 ± 4.23	0.379 + 0.336 + 0.327 = 1.042
	Moderate	94.47 ± 3.99	92.44 ± 4.62	87.30 ± 5.81	89.80 ± 5.29	96.96 ± 3.00	
	Severe	94.47 ± 2.85	96.81 ± 2.19	97.98 ± 1.75	97.39 ± 1.98	90.83 ± 3.59	
	Overall	94.47 ± 2.87	94.45 ± 2.87	94.47 ± 2.87	94.43 ± 2.88	93.25 ± 3.15	

**Table 5:** Two-dimensional CNN five-fold testing performance of severity classifier.

Enhancement	Network	Class	Accuracy	Precision	Sensitivity	F1-Score	Specificity	Inference Time (ms)
Original	VGG 19	Mild	98.77 ± 2.86	98.21 ± 3.44	96.49 ± 4.78	97.34 ± 4.18	99.47 ± 1.88	7.271
		Moderate	94.67 ± 5.55	86.76 ± 8.37	93.65 ± 6.02	90.07 ± 7.39	95.03 ± 5.37	
		Severe	95.90 ± 3.49	97.50 ± 2.75	94.35 ± 4.06	95.90 ± 3.49	97.5 ± 2.75	
		Overall	94.76 ± 2.82	94.89 ± 2.76	94.67 ± 2.82	94.73 ± 2.80	97.32 ± 2.03	
AHE	VGG 19	Mild	98.77 ± 2.86	96.55 ± 4.74	98.25 ± 3.4	97.39 ± 4.14	98.93 ± 2.67	8.161
		Moderate	95.08 ± 5.34	90.48 ± 7.25	90.48 ± 7.25	90.48 ± 7.25	96.69 ± 4.42	
		Severe	96.31 ± 3.32	96.75 ± 3.12	95.97 ± 3.46	96.36 ± 3.3	96.67 ± 3.16	
		Overall	95.08 ± 2.71	95.08 ± 2.71	95.09 ± 2.71	95.08 ± 2.71	97.2 ± 2.07	
Gamma Correction	VGG 19	Mild	88.11 ± 8.40	90.91 ± 7.46	87.72 ± 8.52	89.29 ± 8.03	88.24 ± 8.36	9.651
		Moderate	88.11 ± 7.99	75.71 ± 10.59	84.13 ± 9.02	79.70 ± 9.93	89.50 ± 7.57	
		Severe	88.11 ± 5.70	94.12 ± 4.14	90.32 ± 5.20	92.18 ± 4.73	85.83 ± 6.14	
		Overall	88.11 ± 4.06	88.62 ± 3.99	88.11 ± 4.06	88.28 ± 4.04	87.34 ± 4.17	

## Data Availability

Not applicable.

## Supplementary Materials

The following supporting information can be downloaded at https://www.mdpi.com/article/10.3390/s22114249/s1: Figure S1: heatmap of the correlation matrix with all the features (A) and after removing the highly correlated features (B).
