# Supplementary material for: A Novel Machine Learning Approach for Severity Classification of Diabetic Foot Complications Using Thermogram Images
Source: Sensors (Basel). 2022 Jun 2;22(11):4249. doi: 10.3390/s22114249 (PMC9185274; doi:10.3390/s22114249)
Supplement: Supplementary file 1 [file sensors-22-04249-s001.zip › sensors-1625277-supplementary.pdf]

A

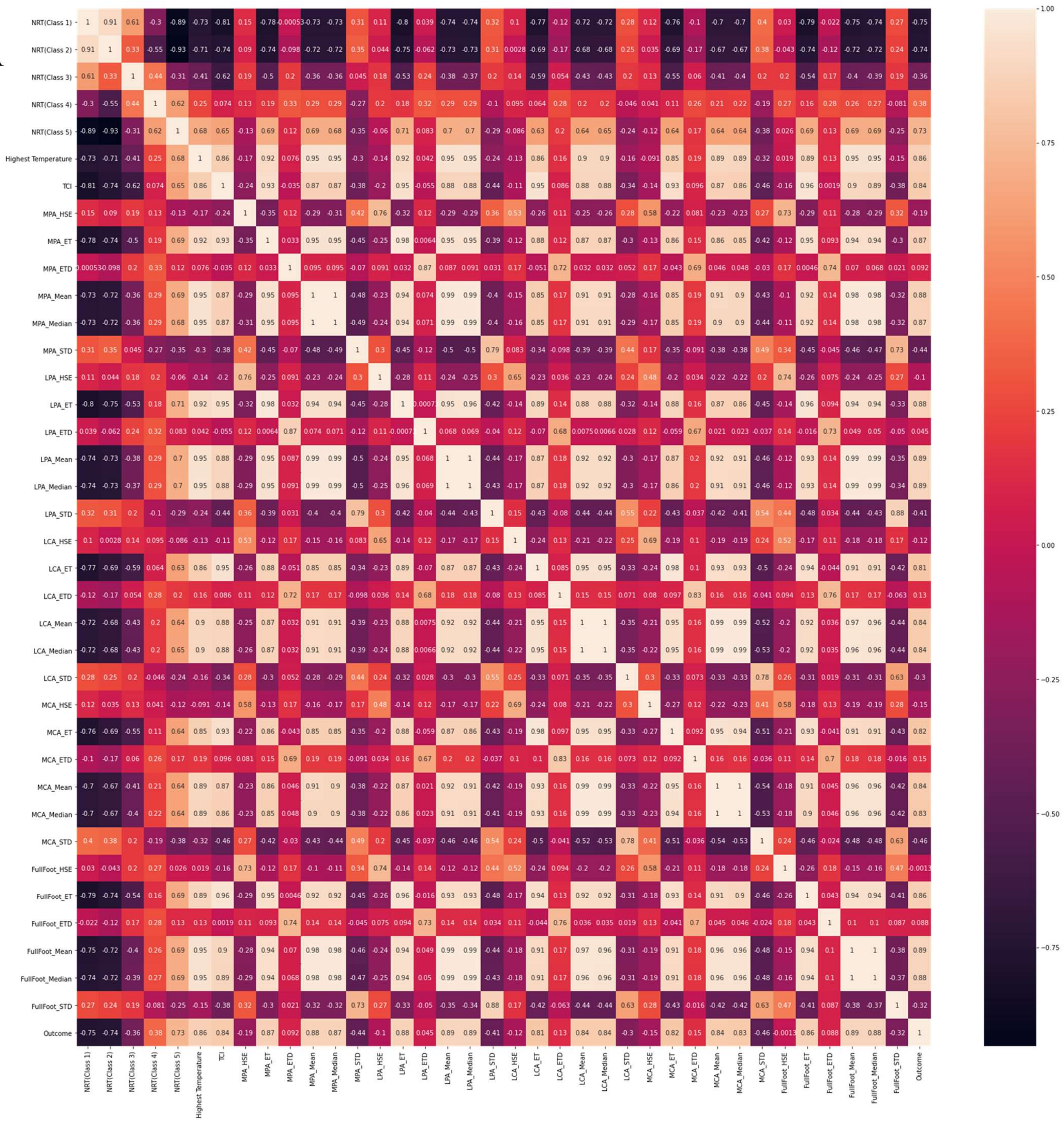
NRT(Class 1)
NRT(Class 2)
NRT(Class 3)
NRT(Class 4)
NRT(Class 5)
Highest Temperature
TCI
MPA_HSE
MPA_ET
MPA_ETD
MPA_Mean
MPA_Median
MPA_STD
LPA_HSE
LPA_ET
LPA_ETD
LPA_Mean
LPA_Median
LPA_STD
LCA_HSE
LCA_ET
LCA_ETD
LCA_Mean
LCA_Median
LCA_STD
MCA_HSE
MCA_ET
MCA_ETD
MCA_Mean
MCA_Median
MCA_STD
FullFoot_HSE
FullFoot_ET
FullFoot_ETD
FullFoot_Mean
FullFoot_Median
FullFoot_STD
Outcome
1.00
0.75
0.50
0.25
0.00
−0.25
−0.50
−0.75

B

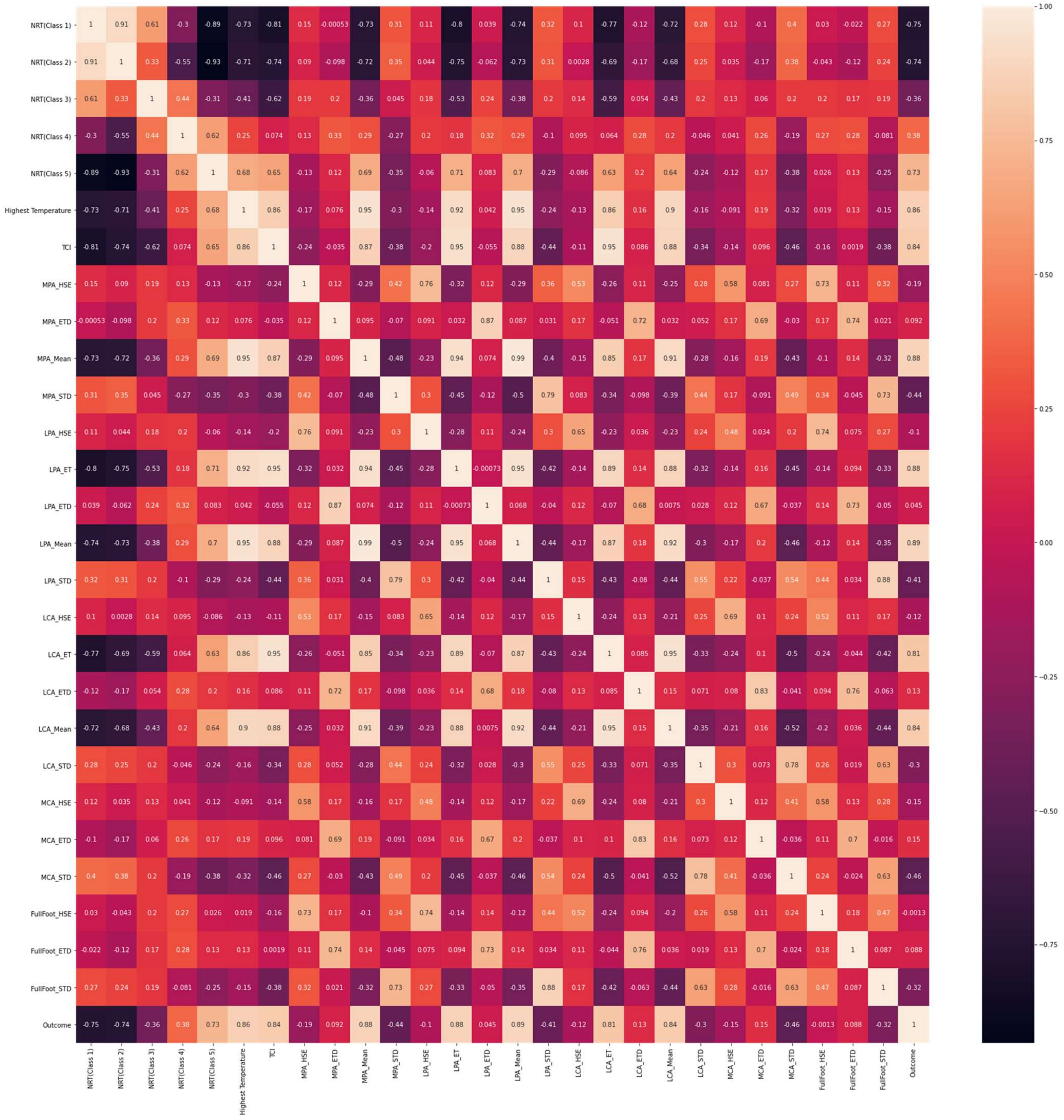

Figure S1. Heatmap of the correlation matrix with all the features (**A**) and after removing the highly correlated features (**B**).
